# Convalescent plasma is of limited clinical benefit in critically ill patients with coronavirus disease-2019: a cohort study

**DOI:** 10.1186/s12967-021-03028-5

**Published:** 2021-08-26

**Authors:** Li Huang, Che Zhang, Xihui Zhou, Zhou Zhao, Weiping Wang, Weidong Leng, Xiao Su, Qizhou Lian

**Affiliations:** 1 The Joint Center for Infection and Immunity, Guangzhou Institute of Pediatrics, Guangzhou Women and Children’s Medical Center, Guangzhou 510623, China; Institute Pasteur of Shanghai, Chinese Academy of Science, Shanghai, 200031 China; 2 Clinical Research Centre, Taihe Hospital, Hubei University of Medicine, Shiyan, China; 3 Intensive Care Unit, The First Affiliated Hospital of Xi’an Jiaotong University, Xi’an, China; 4 Intensive Care Unit, Shiyan People Hospital, Shiyan, China; 5 Department of Respiratory Medicine, Shiyan Xiyuan Hospital, Shiyan, China; 6 Department of Medicine, LKS Faculty of Medicine, The University of Hong Kong, Hong Kong, China; 7 HKUMed Laboratory of Cellular Therapeutics, The University of Hong Kong, Hong Kong, China

**Keywords:** Convalescent plasma, Coronavirus, Coronavirus disease-2019, Mortality, SARS-CoV-2

## Abstract

**Background:**

Recently, convalescent plasma (CP) transfusion was employed for severe or critically ill patients with coronavirus disease-2019. However, the benefits of CP for patients with different conditions are still in debate. To contribute clinical evidence of CP on critically ill patients, we analyze the characteristics and outcomes of patients with or without CP transfusion.

**Methods:**

In this cohort study, 14 patients received CP transfusion based on the standard treatments, whereas the other 10 patients received standard treatments as control. Clinical characteristics and outcomes were analyzed. The cumulative survival rate was calculated by Kaplan–Meier survival analysis.

**Results:**

Data analysis was performed on 24 patients (male/female: 15/9) with a median age of 64.0 (44.5–74.5) years. Transient fever was reported in one patient. The cumulative mortality was 21% (3/14) in patients receiving CP transfusion during a 28-day observation, whereas one dead case (1/10) was reported in the control group. No significant difference was detected between groups in 28-day mortality (*P* = 0.615) and radiological alleviation of lung lesions (*P* = 0.085).

**Conclusion:**

In our current study, CP transfusion was clinically safe based on the safety profile; however, the clinical benefit was not significant in critically ill patients with more comorbidities at the late stage of disease during a 28-day observation.

**Graphic abstract:**

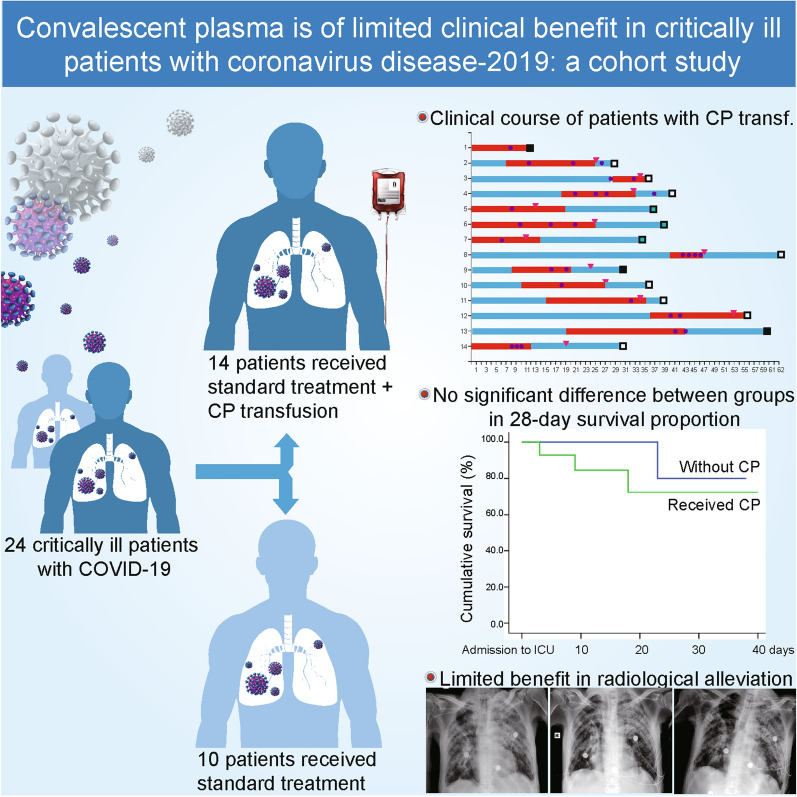

**Supplementary Information:**

The online version contains supplementary material available at 10.1186/s12967-021-03028-5.

## Background

Infection with severe acute respiratory syndrome coronavirus 2 (SARS-CoV-2) has spread worldwide rapidly. SARS-CoV-2 has been identified as the cause of coronavirus disease-2019 (COVID-19) that is considered to be a diverse clade derived from severe acute respiratory syndrome coronavirus (SARS-CoV) and Middle East respiratory syndrome coronavirus (MERS-CoV) [1]. The current treatment strategy mainly relies on antiviral therapy and supportive treatment [2]. Immunotherapies have been applied based on therapeutic plasma exchange [3], mesenchymal stem cell transfusion [4–6], and convalescent plasma (CP) transfusion [7]. Preliminary clinical benefits of CP transfusion have been shown in specific patient populations, including severe [8] or critically ill patients with no more than two comorbidities in some case series [9–12] and case-controlled studies[13]; however, no significant difference was detected in patients with or without CP transfusion based on the 28-day mortality in a randomized clinical trial [14]. The benefits of CP transfusion for patients with different conditions are still in debate. In this cohort study, we present the clinical characteristics and outcomes of 24 critically ill patients with COVID-19, who received treatments with or without CP transfusion.

## Methods

### Study setting, sample size, subjects, and ethical approval

This cohort study set out to evaluate the safety and potential benefit of CP transfusion in critically ill patients with COVID-19. The sample size was calculated using PASS software (version 15.0, NCSS, LLC. Kaysville, Utah, USA). An estimated sample size of 56 (28 patients in a group) would provide 80% power (α = 0.05, two-tail) for an analysis of 28-day mortality, given estimated mortality as 25% in the CP exposed group [15] and 61.5% in the unexposed group [16]. Patients were recruited from four hospitals in West China. Patients in the CP group received the standard treatment with CP according to the criteria defined in the recommendations of the National Health Commission of the People's Republic of China (NHC) [17], who were matched to the control group. The study was reported following the STROBE reporting guideline.

Patients who fulfilled the following criteria were included: (1) diagnosed with laboratory-confirmed COVID-19 according to the World Health Organization (WHO) guideline [18] and the recommendations of the NHC [19]; (2) identified as critically ill according to the recommendations of the NHC [19] (Additional file 1: Table S1); (3) written informed consent was obtained; (4) age ≥ 18 years. Patients who received other investigational treatments were excluded for analysis, including but not limited to mesenchymal stem cell transfusion.

Ethical approval was obtained from the institutional review board of the Affiliated Taihe Hospital of Hubei University of Medicine (Ethical Approval 2020KY04, 2021KS033). Written informed consent was obtained from the patients and/or guardians before data collection.

### Data collection and assessments

Clinical data were retrieved from medical charts by the study team. Data were cross-checked by two researchers for quality control. SARS-CoV-2 RNA was detected by real-time reverse transcription-polymerase chain reaction according to the recommendations of the NHC [20]. A sputum sample or nasopharyngeal/throat swab was taken for testing. Hematological, serum biochemical, and coagulation tests were performed. Chest computed tomography (CT) scans were conducted. In patients with limited mobility, digital radiography was performed at the bedside. Images were stored in picture archiving and communication systems, and reviewed by two experienced radiologists independently. Dissenting opinions between the radiologists were determined by consensus.

### Standard treatments

All patients received antiviral therapy in the form of interferon-α nebulization and arbidol. Antibiotic therapy was prescribed for patients with bacterial pneumonia. Glucocorticoids and mechanical ventilation were initiated if indicated.

### Preparation and transfusion of CP

Donor recruitment, CP collection, quality control, storage, and transportation were performed by Sino Pharm and Wuhan Blood Bank, the specific organizations designated by the NHC in Wuhan. Donors were recruited according to the criteria defined in the recommendations of the NHC [17] and WHO [21] as follows: (1) previously diagnosed with laboratory-confirmed COVID-19; (2) complete resolusion of symptoms at least for 3 weeks before donation; (3) met the criteria for discharge from hospital and quarantine, including negative SARS-CoV-2 RNA test; (4) positive serological test for SARS-CoV-2 antibodies; (5) aged between 18 and 55 years; (6) eligible in the physical exams according to the requirement of national standard operating procedures of blood collection [22], including vital signs and body weight (body weight ≥ 50 kg for male or ≥ 45 kg for female); (7) obtained the information of ABO blood type and eligible in the pretest of blood sample for hemoglobin and alanine aminotransferase; (8) negative in pregnancy test for female donors; (9) negative in HNA and HLA antibody tests for donors with history of gestation or blood transfusion; (10) without a history of transfusion-transmitted infectious disease, and negative for pathogens of transfusion transmitted diseases, including but not limited to hepatitis B virus, hepatitis C virus, human immunodeficiency virus, and syphilis; (11) suitable for donating according to the judgment of physician; (12) written informed consent was obtained.

The plasma from donors was collected and processed by trained staff with the nurse or medical certificates in Sino Pharm and Wuhan Blood Bank, which were certified for routine collection and preparation of blood and plasma in accordance with NHC guidelines [22, 23]. The convalescent plasma was prepared from apheresis collection. Briefly, the plasma was collected in an enclosed system with a blood cell separator, the procedure of which included blood collecting, anticoagulant agent inpouring, blood cell separating, plasma collecting, and blood cells transfusion back to the donor. Essential information was provided on the package of plasma with a unique barcode for source tracking. The plasma was kept at 2–6 °C within 48 h, or freeze at − 20 °C for long-term storage. The plasma samples for release testing were divided from the donated plasma. The quality of CP was checked to meet the following criteria before releasing to clinical centers, including negative for SARS-CoV-2 RNA, hepatitis B virus, hepatitis C virus, human immunodeficiency virus, and syphilis; positive for SARS-CoV-2 specific IgG antibodies in 160 times dilution of serum samples, or positive for SARS-CoV-2 specific total antibodies in 320 times dilution by enzyme-linked immunosorbent assay or chemiluminescence [17]. CP was released to designated treatment centers for clinical use with the approval of the Hubei Provincial Centre for Disease Control and Prevention. CP transfusion was completed within 24 h of receipt following the procedure in the recommendation of NHC [17]. Briefly, patient status was reviewed by physicians to check if any contraindication existed before transfusion, including allergic history to plasma or sodium citrate, or other situation not allowing transfusion. The dosage was calculated according to the bodyweight of the patient 3–5 ml/kg with an upper limitation of 250 ml per dose. Patients were planned to allocate into two sub-groups according to their acute physiology and chronic health evaluation II (APACHE II) scores in a 1:1 ratio to receive a low or high dosage of CP from donors with matched ABO blood types. The patients with less than 10 scores in the evaluation would receive more than 300 ml (< 600 ml) CP in total volume, while the patients with 10 or more scores in APACHE II would receive 600 ml or more (< 900 ml) in total volume. Initial transfusion was conducted in the intensive care unit under close monitoring. The duration of transfusion was over one hour for risk management. Thereafter, transfusion was finished in 40–45 min in patients with good tolerance. The adverse event that occurred during or within 72 h of transfusion was recorded.

### Outcomes

The primary end-point of this study was the mortality of patients in a 28-day observational period. The secondary end-points included radiological alleviation of lung lesions in a 7-day and 28-day period, which was assessed by a CT scoring system for lung lesions [24] (Additional file 1: Table S2). The total scores were calculated as the sum of the scores for five lung lobes. Radiological alleviation was considered when the total scores reduced at least two points or changed to zero. The alleviation rate was analyzed between groups. The cut-off date for survival analysis was 28 days after initial intervention in ICU, except that the patient was discharged or dead before the cut-off date. Patients were discharged if they fulfilled the criteria in recommendations of NHC [19] as follows: (1) fever had recovered for 3 days at least; (2) upper respiratory symptoms were alleviated significantly; (3) the exudative lesion was alleviated remarkably based on radiological images; (4) SARS-CoV-2 nucleic acid was negative in two consecutive tests with an interval of 24 h.

### Statistical analysis

Statistical analysis was performed using SPSS software (version 20.0, International Business Machines Corporation, Armonk, NY, USA). Continuous variables are presented as the median (interquartile range) or mean ± stand error. Categorical variables are presented as number and frequency rates. No imputation was used for missing data. A two-tailed *T*-test was used to compare continuous variables between groups. The Chi-square test or Fisher’s exact test was used to compare categorical variables between groups. The cumulative survival rate was calculated by Kaplan–Meier (K-M) survival analysis. The log-rank test was performed in conjunction with K–M analysis. Statistical significance was considered if *P* < 0.05.

## Results

### Patient characteristics

**Fig. 1:**
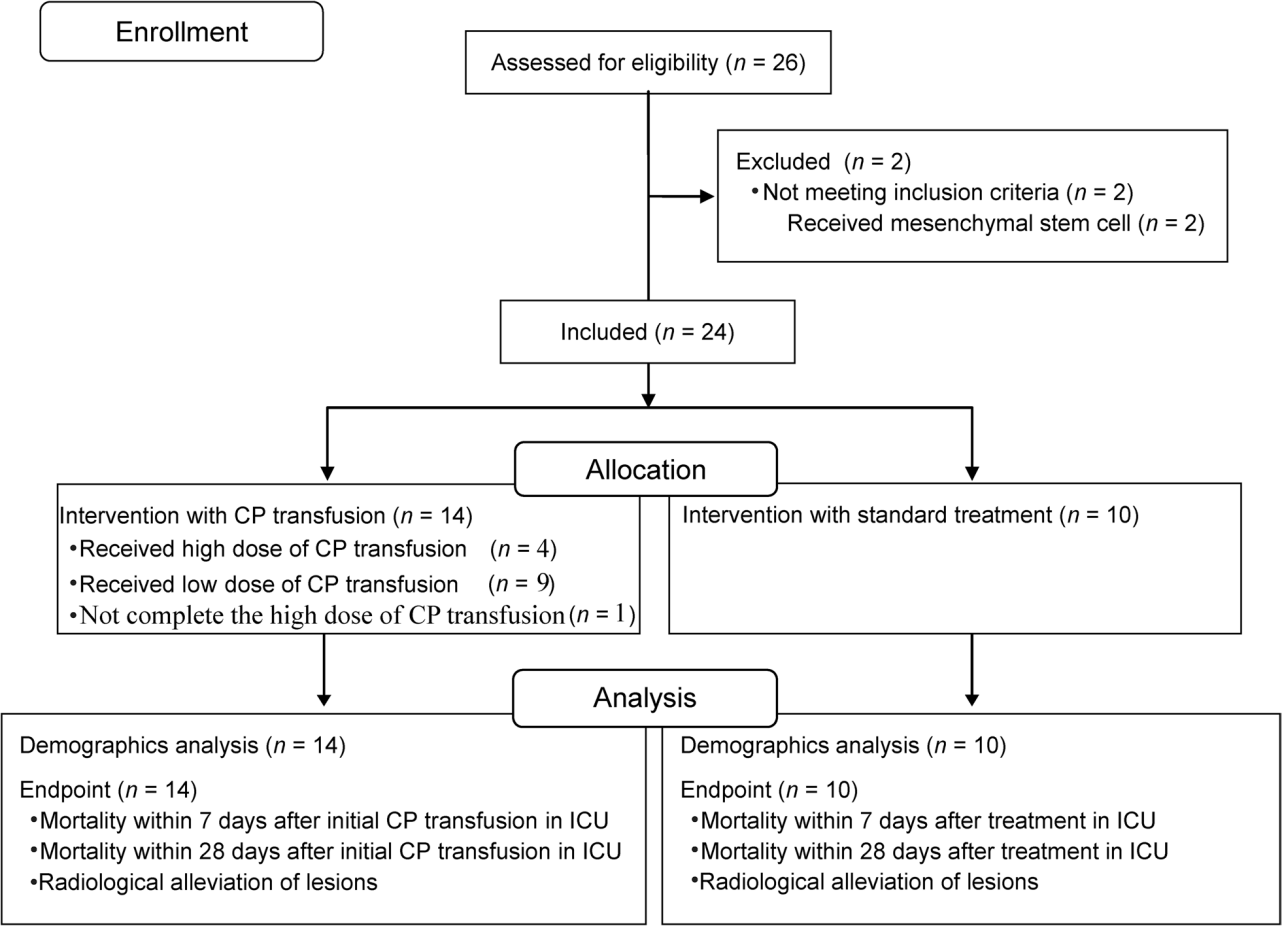
Clinical process and data analysis. Twenty-four patients were included in the study

From January 29 to March 16, 2020, 26 critically ill patients with confirmed SARS-CoV-2 infection were admitted to the hospitals, two of whom were excluded because they had undergone mesenchymal stem cell transplantation (Fig. 1). The recruitment was terminated by the end of March due to the challenge of a limited patient population along with the containment of SARS-CoV-2 infection in West China. Therefore, 24 patients (male/female; 15/9) were enrolled totally with a median age of 64.0 (44.5–74.5) years. The final follow-up visit was completed by April 15, 2020. Notably, all patients had comorbidities, numbering three or more in 12 (50%) patients (Table 1). No significant difference was detected in patient characteristics between groups. A notable decrease of albumin and an increase of high-sensitivity C-reactive protein were detected in patients in both groups. No significant difference was detected in the proportion of abnormal parameters between groups (Additional file 1: Table S3).

**Table 1:** Clinical characteristics of patients (*n* = 24)

Clinical characteristics	Received CP (*n* = 14)	Without CP (*n* = 10)	*P* value
Demographics			
Gender			
Male, *n* (%)	8 (57)	7 (70)	0.678
Female, *n* (%)	6 (43)	3 (30)	0.678
Age, mean ± SE, y	64.6 ± 4.3	55.6 ± 4.8	0.177
< 40 years, *n* (%)	1 (7)	1 (10)	1
40–60 years, *n* (%)	3 (21)	5 (50)	0.204
> 60 years, *n* (%)	10 (71)	4 (40)	0.211
APACHE II score, mean ± SE	12.2 ± 1.6	9.0 ± 0.8	0.121
With three or more comorbidities, *n* (%)	8 (57)	4 (40)	0.680
Main comorbidities			
Chronic obstructive pulmonary disease, *n* (%)	6 (43)	1 (10)	0.171
Diabetes mellitus, *n* (%)	4 (29)	1 (10)	0.358
Hypertension, *n* (%)	4 (29)	1 (10)	0.358
Coronary heart disease, *n* (%)	4 (29)	0	0.114
Main complication			
Bacterial pneumonia, *n* (%)	9 (64)	3 (30)	0.213
Hypoalbuminous edema, *n* (%)	8 (57)	3 (30)	0.240
Respiratory fungal infection, *n* (%)	6 (43)	1 (10)	0.171
Gastrointestinal hemorrhage, *n* (%)	2 (14)	0	0.493

*APACHE* acute physiology and chronic health evaluation, *CP* convalescent plasma, *SE* stand error

### Treatments

**Fig. 2:**
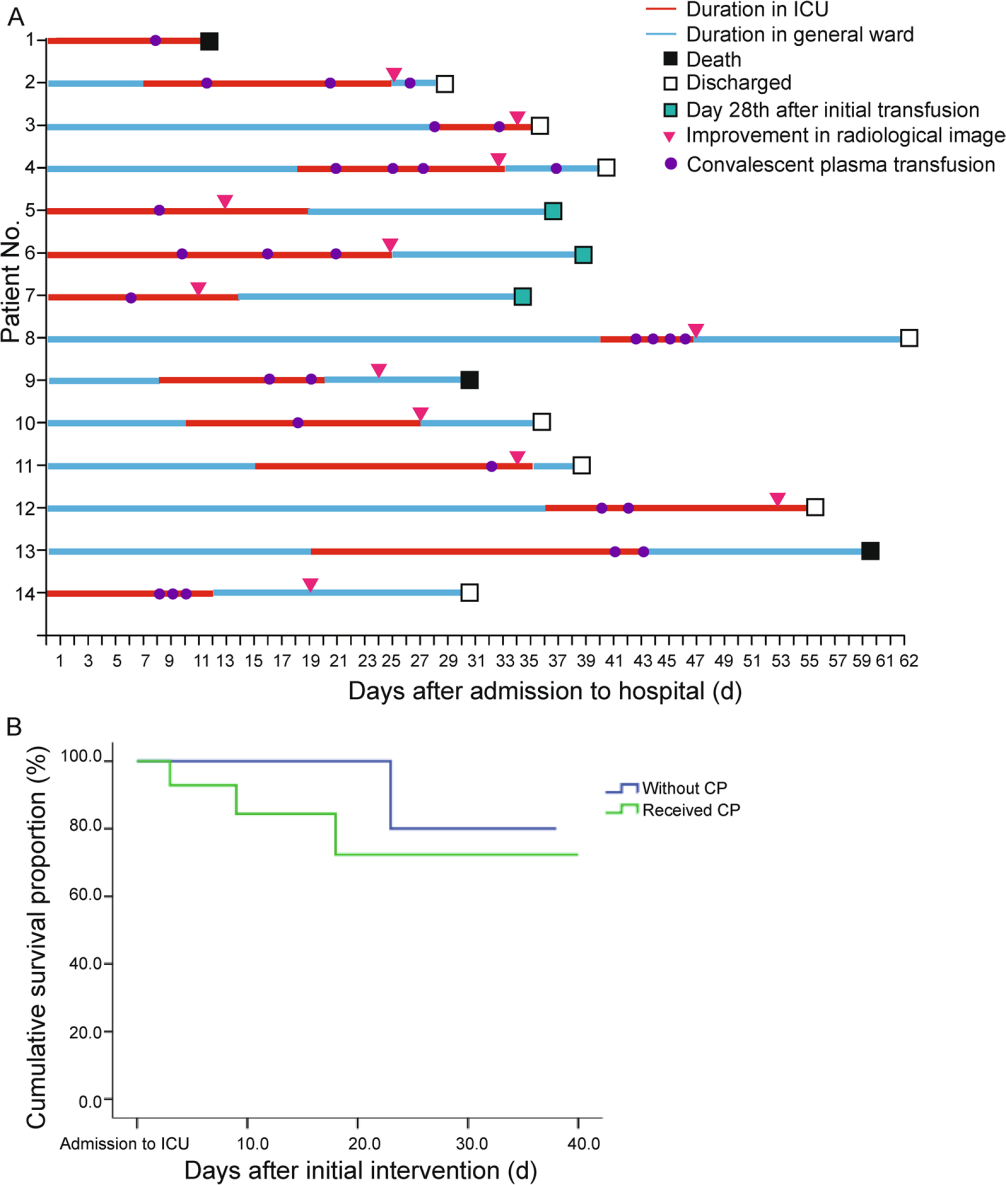
Clinical course of patients in CP transfusion group and cumulative survival analysis. **(A)** Fourteen patients received the initial convalescent plasma transfusion in the intensive care unit. Three patients died within 28 days after the initial transfusion. **(B)** Cumulative survival incidence was calculated by K-M survival analysis. No significant difference was detected between the two groups with or without CP transfusion (Log-rank *P* = 0.321)

All patients received interferon-α nebulization at a dose of 2.5 million IU/ml twice daily and arbidol at 200 mg/dose three times a day according to the recommendation of the NHC [19]. Short-term systemic methylprednisolone was administered to all patients within 3–5 days at a daily dosage of 1–2 mg/kg. Cefoperazone sodium was given to 12 patients (50%) with complicating bacterial pneumonia at a dose of 40 mg/ml twice a day. Caspofungin was administered at a dose of 0.27 mg/ml once a day to seven patients (29%) with fungal infections in the respiratory tract. Mechanical ventilation was required by 12 patients (50%) and extracorporeal membrane oxygenation by one patient. No significant difference was detected between groups in standard treatments. Based on standard treatment, 14 patients received CP transfusion (Fig. 2A) following the recommendations of the NHC [19, 20]. The initial transfusion of CP was conducted at 20.7 (± 3.6) days after hospital admission (Table 2). Finally, five patients were allocated in a high dosage group to receive at least 600 ml of CP in total volume, of whom one patient (Pt. No. 1) was dead before receiving the second dosage due to aggressive progression of the disease. Besides, nine patients received low doses of CP (less than 600 ml), of whom four patients were transmitted from ICU to ordinary wards for relief of fatal symptoms after the first dose of CP (Pt. No. 5, 7, 10, and 11), and did not receive the second doses in accordance with the instruction of NHC [17] (Additional file 1: Table S4).

**Table 2:** Treatments and outcomes of patients (*n* = 24)

Clinical course	Received CP (*n* = 14)	Without CP (*n* = 10)	*P* value
Treatments			
Antiviral, *n* (%)	14 (100)	10 (100)	1
Glucocorticoids, *n* (%)	14 (100)	10 (100)	1
Antibiotics, *n* (%)	9 (64)	3 (30)	0.213
Antifungal, *n* (%)	6 (43)	1 (10)	0.171
Low-flow nasal cannula, *n* (%)	4 (29)	7 (70)	0.095
Mechanical ventilation, *n* (%)	9 (64)	3 (30)	0.214
Extracorporeal membrane oxygenation, *n* (%)	1 (7)	0	1
CP transfusion			
Low dose, *n* (%)	9 (69)	–	–
High dose, *n* (%)	4^a^ (31)	–	–
Initial transfusion time, mean ± SE, d^b^	20.7 ± 3.6	–	–
Mortality			
7-day mortality, *n* (%)^c^	1 (7)	0	1
28-day mortality, *n* (%)^d^	3 (21)	1 (10)	0.615
Radiological alleviation of lesions			
7-day alleviation proportion, *n* (%)^c^	5 (36)	2 (20)	0.653
28-day alleviation proportion, *n* (%)^d^	12 (86)	5 (50)	0.085

*CP* convalescent plasma, *SE* stand error

^a^Patient No. 1 was excluded from the analysis since she could not finish the high-dose therapeutic scheme due to the death of aggressive progression before the second dose

^b^Days from hospital admission to initial transfusion of CP

^c^Within 7 days after initial CP transfusion for patients received CP or treatment without CP in ICU

^d^Within 28 days after initial CP transfusion for patients received CP or treatment without CP in ICU

### Outcomes

A transient fever (37.4 °C) was observed in a patient within 24 h of the first CP transfusion but had resolved by the following day. The cumulative mortality was 21% (three dead cases) in patients who received CP transfusion during a 28-day observational period, whereas one dead case was reported in patients without CP transfusion. However, no significant difference was detected in 28-day mortality (Table 2), and cumulative survival analysis between groups (Fig. 2B, Log-rank *P* = 0.321). Further analysis of the clinical course was performed in dead cases with CP transfusion to identify the underlying reason for poor outcomes. One patient died of multiple organ dysfunction syndromes from extensive disease progression. The other two patients died from deterioration of their comorbidities (Additional file 1: Figure S1), including a cerebral hernia and a relapse of pulmonary tuberculosis respectively. All these events were judged not relative to CP transfusion.

**Fig. 3:**
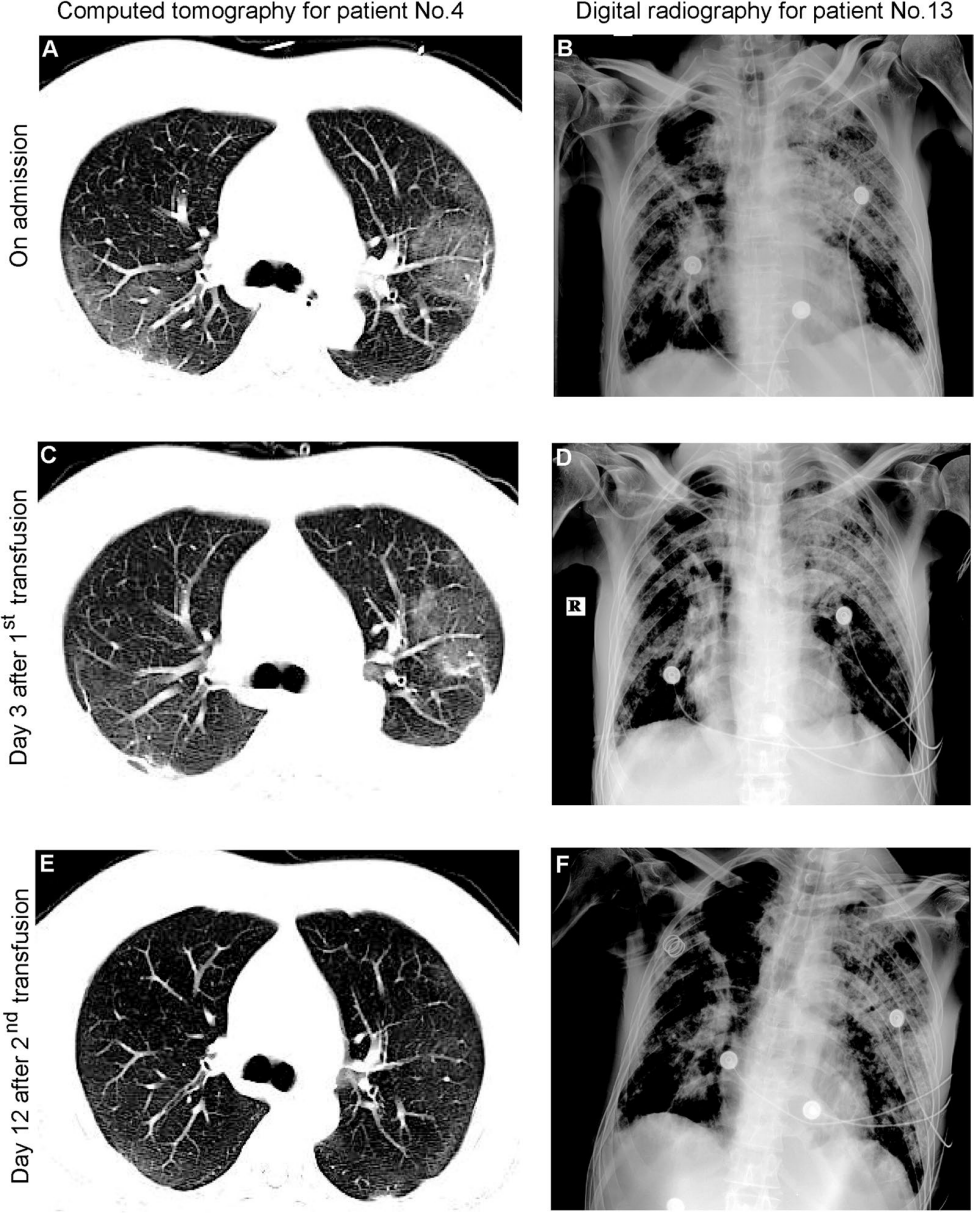
Dynamic changes to chest radiologic images after CP transfusion. **(A)** Ground-glass opacity was observed in bilateral lobules by computed tomography images for patient No.4 on admission to the intensive care unit. **(B)** Patchy shadows of high density were detected in digital radiography for patient No.13 on admission. **(C)** On day 3 after 1^st^ convalescent plasma transfusion, alleviation of lesions was noticed for patient No.4. **(D)** No substantial improvement was detected for patient No.13. **(E)** On day 12 after 2^nd^ convalescent plasma transfusion, notable alleviation was detected for patient No.4. **(F)** No substantial improvement was detected for patient No.13

SARS-CoV-2 RNA became negative for all patients after CP transfusion. Radiological alleviation of lung lesions was detected within 28 days after initial intervention in 12 patients who received CP transfusion and five patients without transfusion, although the difference was not significant statistically (*P* = 0.085, Table 2). Notable improvements were shown in patient No. 4 after CP transfusion (Fig. 3E), who had no severe pulmonary comorbidities and shown better performance in serologic assays for prognosis indicators during the treatment (Additional file 1: Figure S2). No improvement was detected in digital radiography for patient No. 13 (Fig. 3F) who had severe pulmonary comorbidities and poor prognosis according to the serologic assays (Additional file 1: Figure S2).

## Discussion

Currently, the therapeutic strategy for patients with COVID-19 mainly relies on antiviral therapy and supportive treatment. Although some treatments with neutralizing antibodies have shown benefits in preclinical studies [25] and outpatients with COVID-19 [26], the therapeutic potential in critically ill patients with COVID-19 is still under research. As a substitute of monoclonal antibodies for passive immunity, transfusion of CP has been suggested as an option for critically ill patients by the WHO [27] and the NHC [17] based on its empirical utilization in SARS virus infections [28]. Nevertheless, it is very challenging to rescue such critically ill patients who usually died from ARDS or multiple organ failure caused by SARS-CoV-2-provoked destructive immune responses and inflammatory complications. In agreement with previous reports [29], we found that the virus-activated CRS was unlikely to be reversed by CP transfusion, although SARS-CoV-2 RNA did become negative in all patients and alleviation of lung lesions was observed in some patients after CP treatments. Unlike other reports, 12 (50%) patients in our study had three or more comorbidities. Clinical data analysis was performed between patients with or without CP transfusion to evaluate the safety and therapeutic potential of CP in our patient population.

Early safety data were favorable for CP transfusion in a study with 20,000 in-patients with severe or life-threatening COVID-19. Serious adverse events were reported with a low incidence, including transfusion reactions (< 1%), thromboembolic or thrombotic events (< 1%), and cardiac events (around 3%). And the vast majority of the thromboembolic or thrombotic events and cardiac events were judged to be unrelated to CP transfusion [30]. In our study, no serious adverse event was reported. One case of transient fever was reported linked to CP transfusion. It was indicated that CP transfusion would not increase the risk of mortality since no significant difference was detected in the mortality of patients with or without CP transfusion.

The therapeutic efficacy of CP transfusion could be affected by initial transfusion time basing on a study of SARS, which suggested an initial transfusion within 14 days of symptom onset for better outcomes [31]. It was indicated in some case series that severe and critically ill patients could benefit from CP transfusion if given as early as 2 days [32, 33], 3 days [34, 35], 4.5 (± 1.0) days [36], and 13.8 (± 6.5) days after hospitalization [37]. Neutralizing antibodies and the anti-inflammatory effect of CP were indicated to contribute to an attenuation of hypoxia after CP transfusion [38]. In a previous study with a single dose transfusion, the 28-day mortality of critically ill patients was reported to be 28% (8/28) in the CP treatment group, and 35% (10/28) in the control group; however, no significant difference was detected between groups [14]. In a randomized, placebo-control trial, 228 patients with COVID-19 severe pneumonia received CP transfusion at 8 (5–10) days after symptoms onset. No significant differences were observed in clinical status or 30-day mortality between patients treated with (mortality 10.9%) or without (mortality 11.4%) CP [39]. With regards to the patients with moderate COVID-19, it was indicated that CP wasn’t associated with a reduction in progression to severe COVID-19 or all-cause mortality at 28 days after transfusion at 8 (6–11) days upon symptoms onset in an open-label, randomized, controlled trial [40]. It was also indicated in a systematic analysis that the effectiveness of CP treatment was uncertain on all-cause mortality at up to day 28 in patients with moderate and severe COVID-19 [41]. In our study with a repeated transfusion strategy for critically ill patients with more comorbidities, the 28-day mortality was 21% (3/14) in patients with CP transfusion and 10% (1/10) in the control group without CP treatment. In accordance with previous studies, no significant difference was detected between groups, although the initial transfusion was conducted earlier (20.7 ± 3.6 days) than that in a previous study (26 days, IQR 20–36 days) [14].

Critically ill patients could benefit from CP transfusion to achieve pneumonia alleviation in studies with four cases [10] and six cases [11]. However, clinical improvement was not significant between patients with or without CP transfusion within a 28-day observation [14]. In our study, alleviation of lung lesions was evident in 86% (12/14) patients who received CP transfusion; however, the difference could not reach a statistically significant level (*P* = 0.085) between groups with or without CP transfusion. Further study with a larger population was needed to identify the potential of CP on pneumonia alleviation for critically ill patients with COVID-19.

Cytokine release syndrome (CRS) is common in patients with COVID-19 [42] and is believed to be the major cause of morbidity in patients with SARS-CoV and MERS-CoV infection [43]. Recent clinical evidence suggests that the mortality of patients with COVID-19 may be attributed to virus-activated CRS [44]. Increasing levels of interleukin-6, CRP, and other inflammatory cytokines are reported hallmarks of severe MERS-CoV infection [45] and were evident in almost all patients in our study. It is noteworthy that death in our study was not directly related to the progression of pneumonia, but the deterioration of comorbidities. We believe virus-activated CRS may contribute to the deterioration of comorbidities and lead to a poor clinical outcome. According to the laboratory findings in our study, no significant relevance was observed between the serologic assays of several prognosis indicators and CP treatments, including hs-CRP (Additional file 1: Figure S2). The virus-activated CRS was unlikely to be reversed by CP transfusion, although SARS-CoV-2 RNA did become negative in all patients and radiological alleviation of lung lesions was observed in some patients after CP treatments.

## Conclusions

Our findings support the safety of CP transfusion for clinical use. The clinical benefit of convalescent plasma transfusion was not statistically significant in our patient population basing on the data of 28-day mortality, though radiological alleviation of lung lesions was observed in some patients after CP treatments. Although the neutralizing antibody titer level of the CP was controlled by the designated organizations according to the instruction from the NHC, the specific titer for each dose could not be tested due to the limitation of the local laboratory in the early stage of the pandemic. Besides, the interpretation of our findings is limited by the heterogeneous characteristics of patients with different comorbidities and complications at baseline, which failed to adjust due to the small sample size. Also, CP transfusion could not be conducted in an early stage due to diverse patient referrals in different cases and only severe or critically ill patients were recommended for CP treatment according to the instruction of NHC [17]. Further trials with rigorous designs are needed to determine the optimum subject population and therapeutic time window for better clinical benefits.

## Supplementary Information

**Additional file 1: Table S1.** Clinical type of COVID-19. **Table S2.** CT scoring system for lung lesions. **Table S3.** Laboratory findings of patients on admission to the intensive care unit (*n* = 24). **Table S4.** Individual treatment with convalescent plasma transfusion (*n* = 14). **Figure S1.** Comorbidities and complications of patients received convalescent plasma transfusion (*n* = 14). **Figure S2.** Serologic assays for patients received convalescent plasma transfusion (*n* = 14).

## Data Availability

All data generated or analyzed during this study are included in this published article and its Additional file 1.

## Abbreviations

COVID-19: Coronavirus disease-2019
CP: Convalescent plasma
CRS: Cytokine release syndrome
CT: Computed tomography
ICU: Intensive care unit
IQR: Interquartile range
MERS-CoV: Middle East respiratory syndrome coronavirus
NHC: National Health Commission of the People’s Republic of China
SARS-CoV: Severe acute respiratory syndrome coronavirus
SARS-CoV-2: Severe acute respiratory syndrome coronavirus 2
WHO: World Health Organization
