# Supplementary material for: Convalescent plasma is of limited clinical benefit in critically ill patients with coronavirus disease-2019: a cohort study
Source: J Transl Med. 2021 Aug 26;19:365. doi: 10.1186/s12967-021-03028-5 (PMC8390032; doi:10.1186/s12967-021-03028-5)
Supplement: Supplementary file 1 — Additional file 1: Table S1. Clinical type of COVID-19. Table S2. CT scoring system for lung lesions. Table S3. Laboratory findings of patients on admission to the intensive care unit (n = 24). Table S4. Individual treatment with convalescent plasma transfusion (n = 14). Figure S1. Comorbidities and complications of patients received convalescent plasma transfusion (n = 14). Figure S2. Serologic assays for patients received convalescent plasma transfusion (n = 14). [file 12967_2021_3028_MOESM1_ESM.doc]

**Additional file 1**

# Convalescent plasma is of limited clinical benefit in critically ill patients with

# coronavirus disease-2019: a cohort study

# Table S1 Clinical type of COVID-19

| **Clinical type** | **Mild disease** | **Moderate disease** | **Severe disease** | **Critical illness** |
| --- | --- | --- | --- | --- |
| Positive RT-PCR test for SARS-CoV-2 | **+** | **+** | **+** | **+** |
| Upper respiratory symptoms | **+/**– asymptomatic infection | **+** | **+** | **+** |
| Mild pneumonia | – | **+** | **+** | **+** |
| Abnormal radiographic presentation | – | **+** | **+** | **+** |
| Any of manifestations  that suggest disease progression ^†^ | – | – | **+** | **+**  Rapid disease progression |
| Any critical conditions ^‡^ | – | – | – | **+** |

^†^ Manifestations that suggest disease progression:

(1) Rapid breath (≥70 breaths per min for infants aged <1 year; ≥50 breaths per min for children aged >1 year)

(2) Hypoxia

(3) Lack of consciousness, depression, coma, convulsions

(4) Dehydration, difficulty feeding, gastrointestinal dysfunction

(5) Myocardial injury

(6) Elevated liver enzymes

(7) Coagulation dysfunction, rhabdomyolysis, and any other manifestations suggesting injuries to vital organs

^‡^ Critical conditions

(1) Respiratory failure with need for mechanical ventilation (eg, acute respiratory distress syndrome, persistent hypoxia that cannot be alleviated by inhalation through nasal catheters or masks)

(2) Septic shock

(3) Organ failure that needs monitoring in the intensive care unit

Abbreviations: RT-PCR, real-time reverse transcription-polymerase chain reaction; SARS-CoV-2, Severe acute respiratory syndrome coronavirus 2

**Table S2** CT scoring system for lung lesions

| Score | Involvement of lobe |
| --- | --- |
| 0 | None |
| 1 | < 25% |
| 2 | 26% – 49% |
| 3 | 50% – 75% |
| 4 | > 75% |

The total scores were the sum of the scores for five lung lobes.

**Table S3** Laboratory findings of patients on admission to the ICU (*n* = 24)

| **Laboratory parameters** | **Normal range** | **Received CP** | **Without CP** | ***P* value** |
| --- | --- | --- | --- | --- |
|  |  | **(*n* = 14)** | **(*n* = 10)** |  |
| RBC, median (IQR), × 10^12^/ L | 4.0 – 5.5 | 3.4 (3.1 – 3.9) | 3.9 (2.6 – 4.3) |  |
| RBC < 4.0 × 10^12^/ L, *n* (%) |  | 11 (79) | 6 (60) | 0.393 |
| WBC, median (IQR), × 10^9^/ L | 3.5 – 9.5 | 7.2 (5.5 – 9.5) | 5.5 (4.0 – 9.0) |  |
| WBC > 9.5 × 10^9^/ L, *n* (%) |  | 3 (21) | 2 (20) | 1 |
| Platelet count, median (IQR), × 10^9^/ L | 150.0 – 350.0 | 134.5 (94.8 – 276.5) | 149.5 (98.3 – 224.8) |  |
| Platelet count < 150 × 10^9^/ L, *n* (%) |  | 8 (57) | 4 (40) | 0.680 |
| Hematoglobin, median (IQR), g/dl | 110.0 – 160.0 | 101.5 (88.5 – 112.5) | 106.0 (73.5 – 135.8) |  |
| Hematoglobin < 110 g/dl, *n* (%) |  | 9 (64) | 5 (50) | 0.678 |
| Prothrombin time, median (IQR), s | 9.0 – 13.0 | 14.1 (12.5 – 14.8) | 13.5 (12.3 – 19.3) |  |
| Prothrombin time > 13 s, *n* (%) |  | 9 (64) | 4 (40) | 0.408 |
| APTT, median (IQR), s | 25.0 – 37.0 | 33.6 (28.7 – 43.1) | 30.0 (28.9 – 33.9) |  |
| APTT > 37 s, *n* (%) |  | 5 (36) | 1 (10) | 0.341 |
| D-dimer, median (IQR), mg/ L | 0.0 – 0.25 | 0.7 (0.2 – 1.4) | 0.4 (0.1 – 0.7) |  |
| D-dimer > 0.25 mg/ L, *n* (%) |  | 11 (79) | 6 (60) | 0.393 |
| Fibrinogen, median (IQR), g/ L | 2.0 – 4.0 | 4.7 (3.5 – 5.7) | 4.4 (3.4 – 6.3) |  |
| Fibrinogen > 4.0 g/ L, *n* (%) |  | 9 (64) | 5 (50) | 0.678 |
| Albumin, median (IQR), g/ L | 40.0 – 55.0 | 35.4 (31.7 – 39.8) | 38.9 (36.8 – 42.7) |  |
| Albumin < 40 g/ L, *n* (%) |  | 11 (79) | 6 (60) | 0.393 |
| hs-CRP, median (IQR), mg/ L | 0.0 – 5.0 | 49.0 (28.4 – 99.1) | 43.3 (1.9 – 67.5) |  |
| hs-CRP > 5.0 mg/ L, *n* (%) |  | 13 (93) | 7 (70) | 0.272 |
| Creatine kinase, median (IQR), IU/ L | 50.0 – 310.0 | 67.0 (35.3 – 103.4) | 87.0 (70.5 – 173.5) |  |
| Creatine kinase > 310 IU/ L, *n* (%) |  | 2 (14) | 2 (20) | 1 |
| CK-MB, median (IQR), IU/ L | 0.0 – 24.0 | 10.0 (6.8 – 24.7) | 10.5 (7.8 – 15.5) |  |
| CK-MB > 24 IU/ L, *n* (%) |  | 3 (21) | 1 (10) | 0.615 |
| LDH, median (IQR), IU/ L | 100.0 – 240.0 | 278.9 (203.5 – 455.8) | 255.5 (137.5 – 458.8) |  |
| LDH > 240 IU/ L, *n* (%) |  | 9 (64) | 6 (60) | 1 |
| α-DBDH, median (IQR), IU/ L | 72.0 – 182.0 | 212.0 (186.0 – 253.0) | 186.0 (107.0 – 209.0) |  |
| α-DBDH > 182 IU/ L, *n* (%) |  | 10 (71) | 4 (40) | 0.211 |

Abbreviations: APTT, activated partial thromboplastin time; CK-MB, creatine kinase-MB; CP, convalescent plasma; hs-CRP, high-sensitivity C-reactive protein; ICU, intensive care unit; IQR, interquartile range; LDH, lactate dehydrogenase; RBC, red blood cell count; WBC, white blood cell count; α-DBDH, α-hydroxybutyrate dehydrogenase

**Table S4** Individual treatment with convalescent plasma transfusion (*n* = 14)

| **Patient No.** | **Infusion time ^†^** | **Dosage, ml** | **Total volume, ml** |
| --- | --- | --- | --- |
| 1 | Day 8 | 200 | 200 |
| 2 | Day 11 | 200 | 600 |
|  | Day 20 | 200 |  |
|  | Day 26 | 200 |  |
| 3 | Day 28 | 200 | 400 |
|  | Day 33 | 200 |  |
| 4 | Day 21 | 150 | 725 |
|  | Day 25 | 175 |  |
|  | Day 27 | 200 |  |
|  | Day 37 | 200 |  |
| 5 | Day 8 | 150 | 150 |
| 6 | Day 10 | 200 | 550 |
|  | Day 16 | 200 |  |
|  | Day 21 | 150 |  |
| 7 | Day 6 | 150 | 150 |
| 8 | Day 43 | 200 | 825 |
|  | Day 44 | 200 |  |
|  | Day 45 | 200 |  |
|  | Day 46 | 225 |  |
| 9 | Day 16 | 200 | 450 |
|  | Day 19 | 250 |  |
| 10 | Day 18 | 200 | 200 |
| 11 | Day 32 | 200 | 200 |
| 12 | Day 40 | 200 | 400 |
|  | Day 42 | 200 |  |
| 13 | Day 41 | 200 | 375 |
|  | Day 43 | 175 |  |
| 14 | Day 8 | 200 | 600 |
|  | Day 9 | 200 |  |
|  | Day 10 | 200 |  |

^†^ The day after hospital admission.


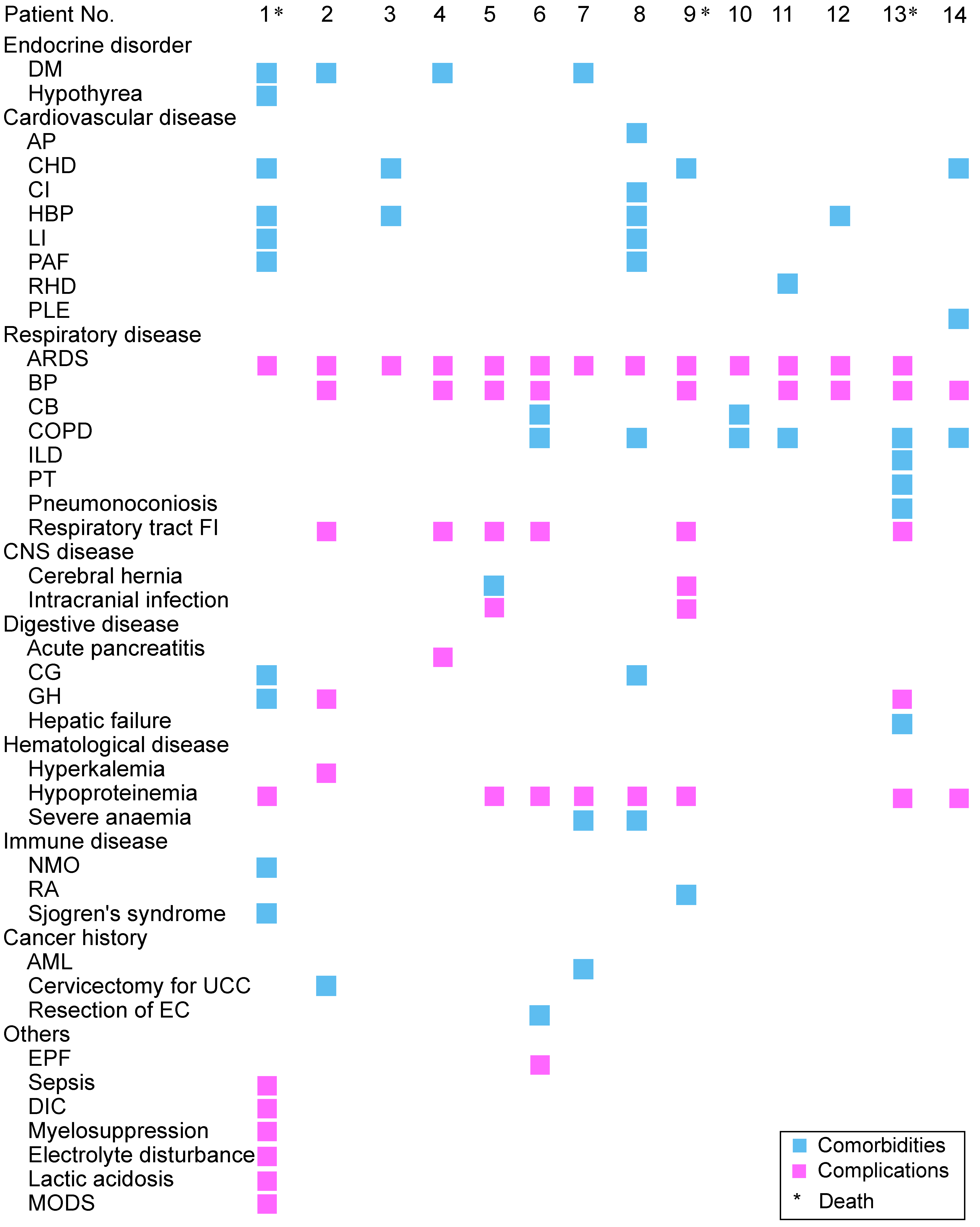


**Fig.S1** Comorbidities and complications of patients (*n* = 14)

The figure shows the comorbidities and complications of the patients. Mixed-infection with respiratory pathogens was detected in patients No. 1, 5, and 7. Among the patients who died, severe comorbidities were particularly noticeable in circulatory and respiratory systems for patients No. 1 and 13, respectively. Cerebral hernia and intracranial infection were noticed in patient No. 9.

Abbreviations: AML, acute myeloid leukemia; AP, angina pectoris; ARDS, acute respiratory distress syndrome; BP, bacterial pneumonia; CB, chronic bronchitis; CG, chronic gastritis; CHD, coronary heart disease; CI, cardiac insufficiency; CNS, central nervous system; COPD, chronic obstructive pulmonary disease; DIC, disseminated intravascular coagulation; EC, esophageal cancer; EPF, esophageal-pleural fistula; FI, fungal infection; GH, gastrointestinal hemorrhage; HBP, hypertension; ILD, interstitial lung disease; LI, lacunar infarction; MODS, multiple organ dysfunction syndrome; NMO, neuromyelitis optica; PAF, paroxysmal atrial fibrillation; PLE, phlebothrombosis of lower extremity; PT, pulmonary tuberculosis; RA, rheumatoid arthritis; RHD, rheumatic heart disease; UCC, uterine cervix cancer.

**
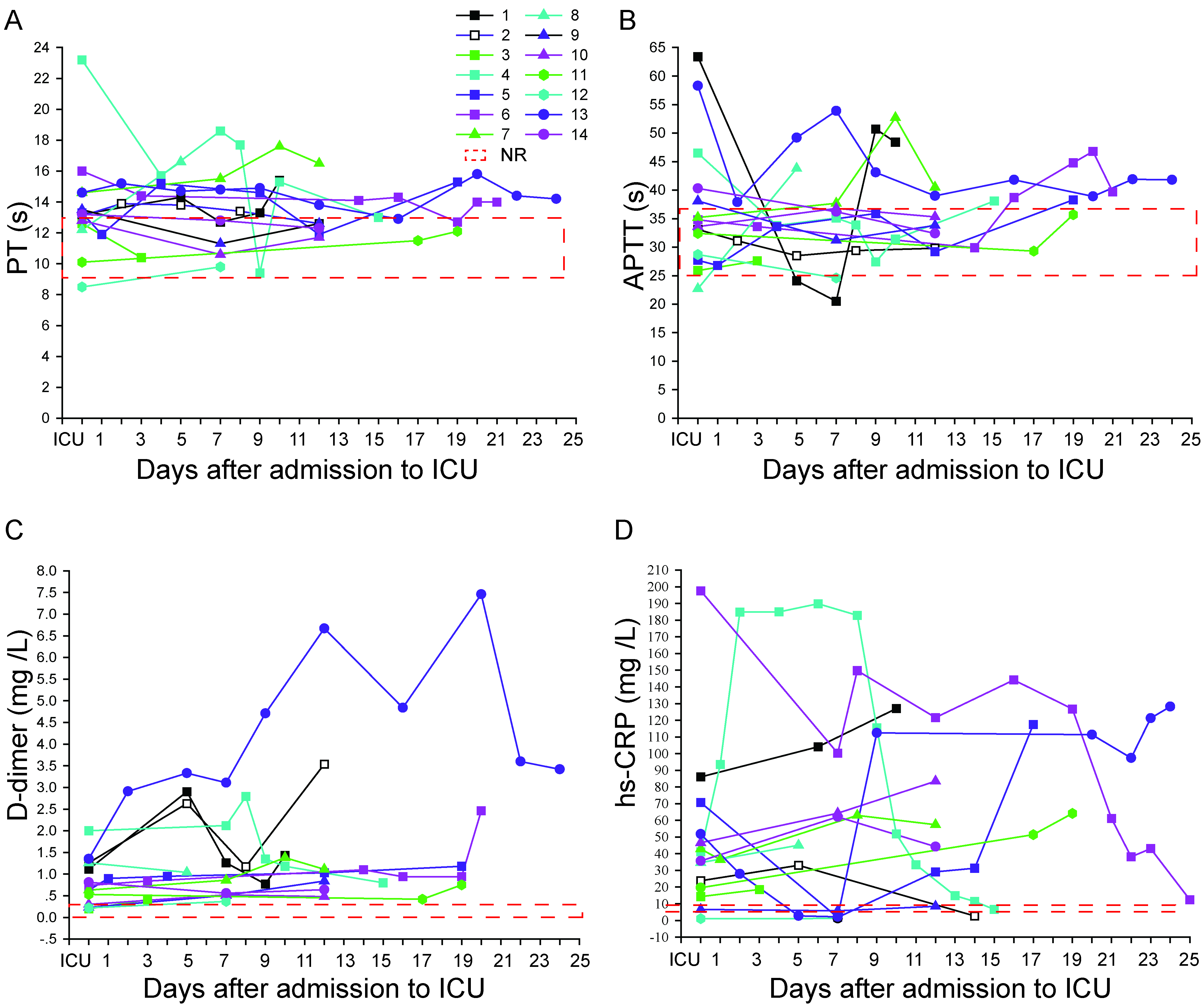
**

**Fig. S2** Serologic assays for patients received convalescent plasma transfusion (*n* = 14)

Several indicators for disease prognosis were detected before and after CP treatments, including PT, APTT, D-dimer, and hs-CRP. No significant relevance was observed between the level of these indicators and CP treatments.

Abbreviations: APTT, activated partial thromboplastin time; hs-CRP, high-sensitivity C-reactive protein; PT, Prothrombin time.
